# Upper Gastrointestinal Bleeding among Patients Admitted in Department of Emergency in a Tertiary Care Centre: A Descriptive Cross-sectional Study

**DOI:** 10.31729/jnma.7409

**Published:** 2022-04-30

**Authors:** Binod Karki, Tshering Wangdi Sherpa, Egesh Aryal, Alisha Adhikari, Binit Upadhaya Regmi, Srijana Katwal, Sujit Kumar Mandol, Rajeeb Kumar Deo, Pravakar Dawadi

**Affiliations:** 1 Department of Medicine, Shree Birendra Hospital, Chhauni, Kathmandu, Nepal; 2 Shree Birendra Hospital, Chhauni, Kathmandu, Nepal; 3 Nepalese Army Institute of Health Sciences, Sanobharyang, Kathmandu, Nepal

**Keywords:** *bleeding*, *emergency medicine*, *upper gastrointestinal tract*, *varices*

## Abstract

**Introduction:**

Upper gastrointestinal bleeding is a common medical emergency with significant morbidity and mortality. Its causes can be classified under variceal bleeding or non-variceal bleeding. Peptic ulcer and variceal bleeding are common causes. Thus, this study aims to find the prevalence of upper gastrointestinal bleeding among patients attending the Department of Emergency in a tertiary care centre.

**Methods:**

This was a descriptive cross-sectional study conducted on patients admitted to the Department of Emergency a tertiary care centre from September 2020 to August 2021 among 3375 patients. The ethical approval was obtained from the Institutional Review Committee of the hospital (Reference number: 328). Patients presenting with the clinical features of upper gastrointestinal bleeding in the form of hematemesis or melena were enrolled after written informed consent. Data entry was done in Statistical Packages for the Social Sciences version 20.0. for descriptive analysis. Point estimate at 95% Confidence Interval was calculated along with frequency and percentage for binary data.

**Results:**

Out of 3375 admissions in the Department of Emergency, 85 (2.52%) (1.99-3.05 at 95% Confidence Interval) patients presented with upper gastrointestinal bleeding.

**Conclusions:**

The prevalence of upper gastrointestinal bleeding is lower in comparison to other studies done in similar settings.

## INTRODUCTION

Upper Gastrointestinal Bleeding (UGIB) is a bleeding proximal to the ligament of Treitz.^1^ It is a medico-surgical emergency requiring frequent hospitalization and mortality ranging from 3-15%.^2,3^

Patients with UGIB present with hematemesis, melena, hematochezia symptoms, suggestive of blood loss or anemia such as light headedness, syncope, angina or occult gastrointestinal bleeding.^4^ The causes of UGIB have been classified into variceal bleeding (esophageal and gastric varices) and non-variceal bleeding (peptic ulcer, erosive gastroduodenitis, reflux esophagitis, tumor, vascular ectasia, etc.), with emergency endoscopy being the standard investigation of choice as it provides both diagnosis and treatment of UGIB.^5^ The peptic ulcer and varices are among its common causes.

The aim of this study is to find the prevalence of UGIB among patients admitted in the Department of Emergency in a tertiary care centre.

## METHODS

This was a descriptive cross-sectional study conducted on patients admitted to the Department of Emergency of Shree Birendra Hospital, Kathmandu, from September, 2020 to August, 2021. The ethical approval was obtained from the Institutional Review Committee of the Nepalese Army Institute of Health Sciences (Reference number: 328). Patients presenting with the clinical features of upper gastrointestinal bleeding in the form of hematemesis or melena were enrolled after written informed consent. Detailed clinical examination was done and duly noted. Patients were subjected to routine laboratory blood investigations and ultrasound of the abdomen. Patients less than 18 years of age, referred from other centres after endoscopy, and not willing to participate were excluded. A convenience sampling was done.

The sample size was calculated by using the formula:

n = (Z^2^ × p × q) / e^2^

  = (1.96^2^ × 0.5 × 0.5) / 0.02^2^

  = 2401

Where,

n = minimum required sample size
Z = 1.96 at 95% Confidence Interval (CI)
p = prevalence taken as 50% for maximum sample size calculation
e = margin of error, 2%

We took 10 % as a non-response rate, thus the final sample size of 2642. However, we took a total of 3375 patients who were admitted to the Emergency Department during the study period.

All patients underwent upper gastrointestinal endoscopy within 24 hours of the admission, either after rapid Coronavirus Disease (COVID-19) antigen or Real-time Polymerase Chain Reaction (Rt-PCR) test wherever available. Standard Personal Protective Equipment (PPE) use and disinfectant measures were taken following each procedure. After identifying the bleeding stigmata, appropriate endotherapy was given as per the requirement. All the participants were treated as per the standard treatment protocol of the hospital. The data was collected and descriptive analysis was done through the Statistical Package for the Social Science (SPSS) software version 20.0. Point estimate at 95% Confidence Interval was calculated along with frequency and percentage for binary data and mean and standard deviation was calculated for continuous data.

## RESULTS

Out of 3375 admissions in the Department of Emergency, 85 (2.52%) (1.99-3.05 at 95% Confidence Interval) patients presented with upper gastrointestinal bleeding.

The most common findings among UGIB patients were varices 40 (47.1%) followed by peptic ulcer disease 21 (24.7%) (Table 1).

**Table 1:** Findings among UGIB patients (n= 85).

Findings	n (%)
Varices	40 (47.1)
Peptic ulcer disease	21 (24.7)
Gastric erosions	11 (12.9)
Gastric growth	5 (5.9)
Mallory weiss tear	8 (9.4)

Among the 85 UGIB patients, the mean age of the population was 58.44±13.05. Most of the patients, 46 (54.10%), were in the age group 41-60, followed by 34 (40%) above 60 years of age. Out of 85 patients, 65 (76.50%) were male (Table 2).

**Table 2:** Age group of participants (n= 85).

Age group	n (%)
Less than 40	5 (5.88)
41-60	46 (54.12)
More than 60	34 (40.00)

Out of 21 cases of peptic ulcer disease, the endoscopic diagnosis of the bleeding lesion as done by Forrest classification indicated 19 (90.5%) cases as Forrest III (Table 3).

**Table 3:** Forrest group classification of peptic ulcer related UGIB (n= 21).

Forest group	n (%)
Forrest Ib	1 (4.76)
Forrest IIb	1 (4.76)
Forrest III	19 (90.48)

Amongst patients with UGIB, hematemesis and melena were the commonest presentations with 41 (48.24%) followed by melena only in 23 (27.06%), and hematemesis only in 18 (21.18%). Syncope was present in 25 (29.41%), dizziness in 15 (17.65%), and generalized weakness in 18 (21.18%). On clinical examination, splenomegaly was present in 13 (15.30%), hepatomegaly in 8 (9.41%), and ascites in 23 (27.06%). Pallor was evident in 50 (58.82%) and jaundice in 23 (27.06%) patients. Laboratory evaluation revealed mean hemoglobin of 8.3±3.17 g/dl and a mean International Normalized Ratio (INR) of 1.48±0.85.

All patients were resuscitated as per the standard protocol. All patients received bolus pantoprazole infusion followed by a maintenance infusion. In addition, those patients who were known to have chronic liver disease from their previous records or with clinical examinations, as well as imaging and laboratory findings suggestive of chronic liver disease, were given octreotide or terlipressin as per the physician's advice. All patients underwent upper GI endoscopy within 24 hours of admission. Endotherapy was required in 43 (50.59%) cases, which included esophageal band ligation in 40 (47.06%) cases followed by injection adrenaline and heater probe in two duodenal ulcers, and hemoclips application in two bleeding cases of peptic ulcer disease and one large Mallory Weiss tear. Blood transfusion was required in 49 (57.65 %) cases, and the median amount of transfusion was 2 (0-2) pint. Most of the patients, 34 (40%), required up to two pints of blood, and 11 (12.94%) patients required four or more pints of blood.

## DISCUSSION

In our study, the prevalence of upper gastrointestinal bleeding among patients admitted in the Department of Emergency was seen as 2.52%. The study was conducted during the time of the Coronavirus Disease-19 (COVID-19) pandemic. Although the percentage prevalence was not mentioned in similar studies conducted in Nepal, our study found a lower number of UGIB cases.^4,6-8^ This may be due to reduced patient visits to the hospital due to various forms of COVID-19 lockdown implemented by the government during the study period and public concerns on the transmission of Severe Acute Respiratory Syndrome Coronavirus 2 (SARS-CoV-2) from the hospital.

Our study showed that the prevalence of UGIB is more in the age group of 40-60 years, with the mean age being 58±13 years; however, the occurrences in age >60 are also substantial. These findings may be due to concurrent risk factors in subjects of age group 4060 and higher. Data from various studies conducted in Nepal showed that the mean age of patients was 49±12.64, 49±12.64, and 51.6 years, respectively, which are in line with our study.^6,9,10^ However, a study displayed UGIB in the younger age group with the mean age being 45.32±18.47.^4^

Manifestations of UGIB can be either obvious or hidden. The observations can vary in different studies. Hematemesis and melena were the most common presentation (48.2%) in our study, followed by melena alone (27%) and hematemesis alone (21%). In contrast, the study revealed that 71.7% presented with hematemesis and melena both, 20% melena only and 8.3% hematemesis only.^8^

In our study, varices are found to be the most common findings among the patients with UGIB (47.1%) followed by gastric and duodenal ulcer (24.7%), gastric erosions (12.9%), Mallory Weiss tear (9.4%) and gastric malignancy (5.9%). Similar pattern of findings was observed in a study in 2013 where variceal bleeding were observed in 33.1%, peptic ulcers in 21.9%, gastric erosion in 12.1% and Mallory Weiss tear in 2.5%.^10^ However, other studies found peptic ulcer disease as common findings followed by variceal bleeding among patients with UGIB.^4,7,9^ This may be due to difference in socio-economic and demographic difference between the local population where the study was conducted.

The prognosis of peptic ulcer bleeding widely depends upon rebleeding and recurrence of hemorrhage. Forrest classification is used in patients with peptic ulcer disease for risk assessment prediction of rebleeding and can be widely applied to guide endoscopic hemostasis.^11^ Our study revealed a clean base (Forrest III) to be present in 90% of cases, 5% with adherent clot (Forrest II b) and 5% had oozing hemorrhage (Forrest Ib). According to a study, 4.2% had oozing hemorrhage, 13.6% had an adherent clot, 35.1% had hematin on ulcer base and the rest 47.1% had clean ulcer base and were classified as Forrest IB, IIB, IIC and III, respectively.^9^ A study showed maximum number of the patients (70%) with peptic ulcer disease had ulcers with hematin base (Forrest IIC).^8^

This study had several limitations. Data for the study was taken from a single center. Our study period was during the lockdown phase, so the actual prevalence of UGIB in a community is likely to be more than recorded. Moreover, gastroenterologists may have been reluctant to do an endoscopy on clinically stable patients, contributing to fewer cases. However, a change in the pattern of UGIB during the COVID-19 pandemic can be reflected in the study.

## CONCLUSIONS

The prevalence of upper gastrointestinal bleeding is lower in comparison to other studies done in similar settings. Upper gastrointestinal bleeding is a common medical emergency requiring immediate endoscopic assessment.

## References

[1] Fallah MA, Prakash C, Edmundowicz S (2000). Acute gastrointestinal bleeding.. Med Clin North Am..

[2] Laine L, Yang H, Chang SC, Datto C (2012). Trends for incidence of hospitalization and death due to GI complications in the United States from 2001 to 2009.. Am J Gastroenterol..

[3] Hearnshaw SA, Logan RF, Lowe D, Travis SP, Murphy MF, Palmer KR (2011). Acute upper gastrointestinal bleeding in the UK: patient characteristics, diagnoses and outcomes in the 2007 UK audit.. Gut..

[4] Gurung RB, Joshi G, Gautam N, Pant P, Pokhrel B, Koju R (2010). Upper gastro-intestinal bleeding: aetiology and demographic profile based on endoscopic examination at Dhulikhel Hospital, Kathmandu University Hospital.. Kathmandu Univ Med J (KUMJ)..

[5] Pongprasobchai S, Nimitvilai S, Chasawat J, Manatsathit S (2009). Upper gastrointestinal bleeding etiology score for predicting variceal and non-variceal bleeding.. World J Gastroenterol..

[6] Paudel MS, Kc S, Mandal AK, Poudyal NS, Shrestha R, Paudel BN (2017). Acute Upper Gastrointestinal Bleeding in a Tertiary Care Centre of Nepal.. J Nepal Med Assoc..

[7] Bhattarai J, Acharya P, Barun B, Pokharel S, Uprety N, Shrestha NK (2007). Comparison of endoscopic findings in patients from different ethnic groups undergoing endoscopy for upper gastrointestinal bleed in eastern Nepal.. Nepal Med Coll J..

[8] Dewan KR, Patowary BS, Bhattarai S (2014). A study of clinical and endoscopic profile of acute upper, gastrointestinal bleeding.. Kathmandu Univ Med J (KUMJ)..

[9] Bhattarai S (2020). Clinical Profile and Endoscopic Findings in Patients with Upper Gastrointestinal Bleed Attending a Tertiary Care Hospital: A Descriptive Cross-sectional Study.. J Nepal Med Assoc..

[10] Shrestha UK, Sapkota S (2014). Etiology and adverse outcome predictors of upper gastrointestinal bleeding in 589 patients in Nepal.. Dig Dis Sci..

[11] de Groot NL, van Oijen MG, Kessels K, Hemmink M, Weusten BL, Timmer R (2014). Reassessment of the predictive value of the Forrest classification for peptic ulcer rebleeding and mortality: can classification be simplified?. Endoscopy..

